# Efficient Fluoride Removal Using a CeO_2_/Attapulgite (ATP) Composite

**DOI:** 10.3390/nano15050357

**Published:** 2025-02-26

**Authors:** Jianguo Zhu, Yeting Chen, Xin Xiao

**Affiliations:** 1 Institute of Pharmaceutical and Biomaterials, Lianyungang Normal College, Lianyungang 222006, China; zjgzz117@163.com (J.Z.); 13851266965@163.com (Y.C.); 2 Jiangsu Key Laboratory of Function Control Technology for Advanced Materials, Jiangsu Ocean University, Lianyungang 222005, China

**Keywords:** fluoride removal, adsorption, hydrothermal synthesis

## Abstract

In this study, a CeO_2_/attapulgite (ATP) composite was synthesized via a straightforward hydrothermal method to efficiently remove excess fluoride from water. The structural and surface properties of the synthesized adsorbent were systematically characterized using scanning electron microscopy (SEM), transmission electron microscopy (TEM), X-ray diffraction (XRD), and X-ray photoelectron spectroscopy (XPS). The fluoride adsorption capacity of the CeO_2_/ATP composite was systematically evaluated, reaching a maximum of 47.84 mg/g. Kinetic analysis revealed that fluoride uptake followed a pseudo-second-order model, suggesting a chemisorption-dominated process. Furthermore, isothermal adsorption experiments conducted within a concentration range of 10 mg/L to 260 mg/L demonstrated that the adsorption process fit the Langmuir isotherm model. To evaluate the potential for commercial use, five consecutive reusability tests were performed, showing a sustained adsorption capacity of 30.2 mg/g. The CeO_2_/ATP composite demonstrates effective fluoride removal capabilities and good recyclability, highlighting its potential for practical applications in water treatment.

## 1. Introduction

Rapid industrialization worldwide has led to a substantial rise in industrial wastewater discharge, exacerbating water pollution and posing serious environmental challenges [1]. In particular, the release of fluoride-containing wastewater poses substantial risks to human health and causes extensive damage to environmental ecosystems [2]. Fluoride is an essential trace element that supports the development of teeth and bones [3]; however, excessive concentrations in wastewater pose significant health risks, including skeletal and dental fluorosis [4]. According to the World Health Organization (WHO) guidelines, the fluoride concentration in drinking water should not exceed 1.5 mg/L. In contrast, China enforces a more stringent standard at 1.0 mg/L. Alarmingly, over 25 countries, home to approximately 2.7 billion people, are currently consuming drinking water with fluoride levels that exceed these limits [5]. This situation underscores the urgent need for the development of effective technologies to remove excess fluoride from drinking water.

The removal of fluoride from water presents a complex and challenging process. Currently, established methods for fluoride removal include chemical precipitation [6], electrodialysis [7], ion exchange [8], membrane filtration [9], and adsorption [10]. However, chemical precipitation is hindered by the presence of magnesium and phosphate ions, and the effectiveness of reagent addition diminishes when ammonia nitrogen reaches critical concentrations, leading to increased operational costs. Consequently, chemical precipitation is often employed alongside other advanced treatment methods [11]. Electrodialysis systems typically utilize plastic components, which may suffer from aging, resulting in higher maintenance costs. Additionally, electrodialysis performance is directly influenced by the quality and volume of the feed water, resulting in unstable operational efficiency [12]. Ion exchange is another widely used method. However, it is the most costly approach, and the presence of competing ions can inhibit fluoride removal [13]. Moreover, the resin regeneration process generates a substantial amount of fluoride waste. Membrane separation technology, while offering good selectivity, has inherent limitations that preclude effective separation of certain substances [14]. Among these fluoride removal strategies, the use of new adsorptive materials presents several advantages, including simplicity, cost-effectiveness, sustainability, and high efficiency. The performance and industrial applicability of adsorbent materials are primarily determined by their high surface area [15], selectivity [16], chemical stability [17], and low cost [18], highlighting the urgent need for the development of adsorbents that fulfill these critical requirements.

Attapulgite (ATP) is a magnesium-rich hydrated aluminosilicate clay mineral featuring a stratified chain-like structure, classified as a specialized non-metallic material [19]. Comprising a double-chain Si-O tetrahedral structure and an Al-O octahedral framework, ATP exhibits a high surface area and notable ion-exchange capacity. Owing to its excellent adsorption performance, cost-effectiveness, and widespread availability, ATP has been extensively employed for the removal of excess metal compounds from water [20]. However, the high value of rare earth elements and various natural factors may restrict its effectiveness in groundwater fluoride removal [21]. There is an urgent need to develop a low-cost adsorbent with high fluoride removal capacity across a wide range of conditions. Cerium, which is abundant in the Earth’s crust, makes CeO_2_ a cost-effective and readily available rare earth metal oxide. The ease of synthesizing CeO_2_, combined with its superior adsorption performance and selectivity for fluoride ions, renders it a promising candidate as a selective adsorbent. Importantly, nanoscale CeO_2_ demonstrates enhanced performance relative to its bulk form due to its increased surface area and greater number of available adsorption sites. A considerable body of research has been devoted to improving the fluoride removal efficiency of CeO_2_ nanomaterials.

**Figure 1:**
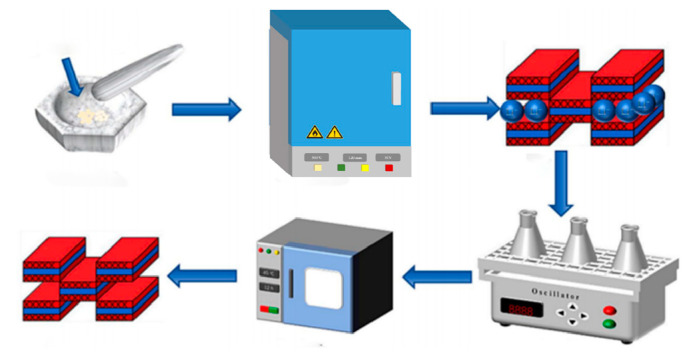
Schematic diagram of the preparation process of CeO_2_/ATP.

In this study, a CeO_2_/attapulgite (ATP) composite was synthesized by integrating rare-earth elements with the cost-effective metal cerium to evaluate its effectiveness in fluoride adsorption from aqueous solutions. The effects of dosage, pH, and coexisting ions on fluoride removal efficiency were systematically investigated. Characterization techniques, including X-ray photoelectron spectroscopy (XPS) and energy-dispersive X-ray spectroscopy (EDS), revealed that fluoride ion capture and removal by the CeO_2_/ATP adsorbent occurs primarily through a combination of physical and chemical adsorption mechanisms. Experiments conducted with real water samples confirmed the fluoride removal capability of the CeO_2_/ATP composite under various environmental conditions. Based on these experimental findings and characterization data, the CeO_2_/ATP composite demonstrates significant potential as an efficient fluoride removal agent in water treatment applications (Figure 1). This modification provides a smooth transition between the two segments, ensuring that the introduction flows logically while conveying the necessary information about the materials and methods employed.

## 2. Adsorption Tests

A sodium fluoride (NaF) stock solution was prepared by dissolving 0.221 g of NaF in 1 L of ultrapure water and subsequently diluted to obtain standard solutions of varying concentrations. Adsorption experiments were conducted in 100 mL conical flasks, each containing 50 mL of fluoride solution at predetermined concentrations. A 50 mg sample of the adsorbent—either ATP, CeO_2_, or CeO_2_ modified with different amounts of ATP (0.1 g red ATP, 0.1 g white ATP, 0.15 g red ATP, 0.2 g red ATP, or 50 mg red ATP)—was introduced into each flask. A 50 mL aliquot of a 10 mg/L NaF solution was measured using a graduated cylinder and added to each flask, followed by the placement of a stir bar. The mixtures were continuously stirred on a magnetic stirrer for 2 h, with fluoride ion concentrations monitored at 20 min intervals until adsorption equilibrium was reached. The adsorption capacity was then calculated.

Kinetic adsorption experiments were performed, and the collected data were analyzed using pseudo-first-order, pseudo-second-order, and intra-particle diffusion models. Adsorption isotherms were established for initial NaF concentrations ranging from 10 to 260 mg/L. To investigate the effect of pH on fluoride removal efficiency, the solution pH was adjusted using 0.1 mol/L hydrochloric acid (HCl) or sodium hydroxide (NaOH). Cyclic regeneration experiments were conducted using 0.5 mol/L NaOH and 0.5 mol/L sodium carbonate (Na_2_CO_3_) solutions for desorption. After five regeneration cycles, the adsorption capacity was reassessed to determine the reusability of the adsorbent. The adsorption capacity (q_e_, mg/g) was calculated using the following equation:

$$\frac{\left(c_{0}-c_{e}\right)V}{m}$$

where C_0_ (mg/L) and C_e_ (mg/L) are the initial and equilibrium fluoride concentrations, respectively; V (L) is the solution volume, and m (g) is the mass of the adsorbent.

## 3. Results and Discussion

### 3.1. XRD Analysis

**Figure 2:**
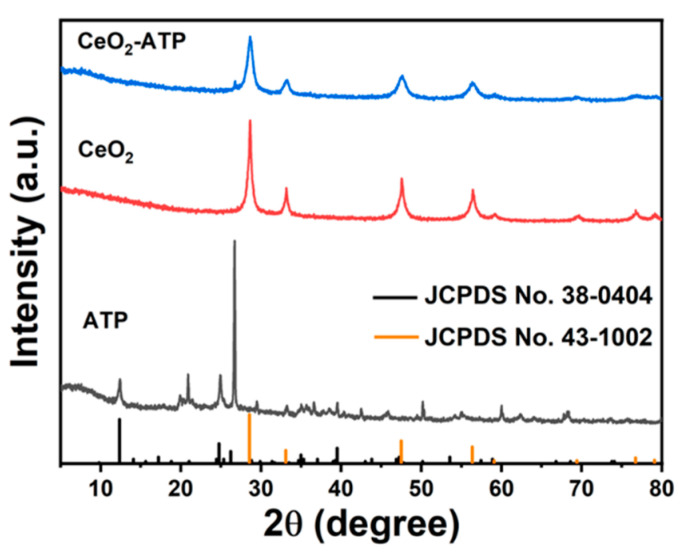
XRD spectra of ATP, CeO_2_, CeO_2_/ATP.

X-ray diffraction (XRD) analysis was conducted on attapulgite (ATP), CeO_2_, and the CeO_2_/ATP composite, as depicted in Figure 2. The XRD pattern of CeO_2_ corresponds to JCPDS No. 43-1002, while ATP corresponds to JCPDS No. 38-0404. The principal diffraction peaks for CeO_2_ are observed at 2θ values of 28.6°, 33.1°, 47.5°, 56.3°, 59.1°, 69.4°, 76.7°, and 79.1°, which correspond to the crystal planes (111), (200), (220), (311), (222), (400), (331), and (420), respectively. The principal diffraction peaks for ATP are observed at 2θ values of 9.809°, 24.745°, 25.332°, 35.321°, 39.491°, 68.588°, which correspond to the crystal planes (4 0 0), (10 0 0), (8 0 1), (−12 0 2), (14 0 1), and (−14 0 5), respectively. At the same time, the CeO_2_/ATP pattern shows not only the diffraction peaks of CeO_2_ but also some of the characteristic peaks of ATP. These results certify the success synthesis of both the CeO_2_ and CeO_2_/ATP materials.

#### 3.1.1. SEM Analysis

**Figure 3:**
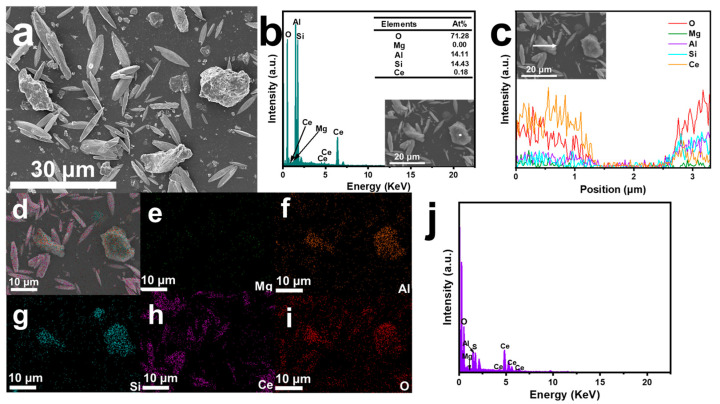
CeO_2_/ATP of (**a**) SEM images; (**b**) point-scan spectra; (**c**) linear-scan spectra; (**d**) elemental mapping spectra of (**e**) Mg, (**f**) Al, (**g**) Si, (**h**) Ce; and (**i**) O, (**j**) EDS energy spectra.

In Figure 3a, the modified ATP showed a structure that incorporated spindle-shaped CeO_2_ within a block-like ATP framework, thereby verifying the successful integration of the two materials and the even distribution of CeO_2_ throughout the composite.

Figure 3b illustrated a spot scan of a selected area, revealing that the primary elements in this region were Si, Mg, and O, suggesting that ATP was the dominant component. The line scan presented in Figure 3c indicated the presence of five distinct elements, further verifying that the CeO_2_/ATP compound was successfully synthesized. Finally, Figure 3e–i provided elemental mapping and energy-dispersive spectroscopy (EDS) spectra, demonstrating that all elements were evenly distributed across the surface of the CeO_2_/ATP composite. Collectively, these findings provided robust evidence for the successful synthesis of the CeO_2_/ATP composite.

Figure S1a,b presented pictures from scanning electron microscopy (SEM) of Attapulgite (ATP) at different magnifications. The images revealed that ATP exhibited a rod-like crystal structure. Figure S1d–g displayed the elemental distribution maps for ATP, as indicated in Figure S1c. The elements O, Si, Mg, and Al were uniformly distributed throughout the ATP structure. The elemental distribution maps indicated a high content of O, followed by Si, along with the presence of metallic elements Al and Mg. This composition closely matched the ideal chemical formula for ATP, Mg_5_Si_8_O_20_(OH)_2_·4H_2_O, which often contained additional cations such as Na^+^, Ca^2+^, Fe^3+^, and Al^3+^ in its crystal structure.

Figure S2a,b displayed the SEM images of cerium dioxide (CeO_2_) at varying magnifications, illustrating a spindle-shaped morphology. Figure S2c–f provided elemental mapping for CeO_2_, where Ce and O elements were uniformly distributed across the material’s surface, certifying the successful synthesis of CeO_2_.

#### 3.1.2. TEM Analysis

**Figure 4:**
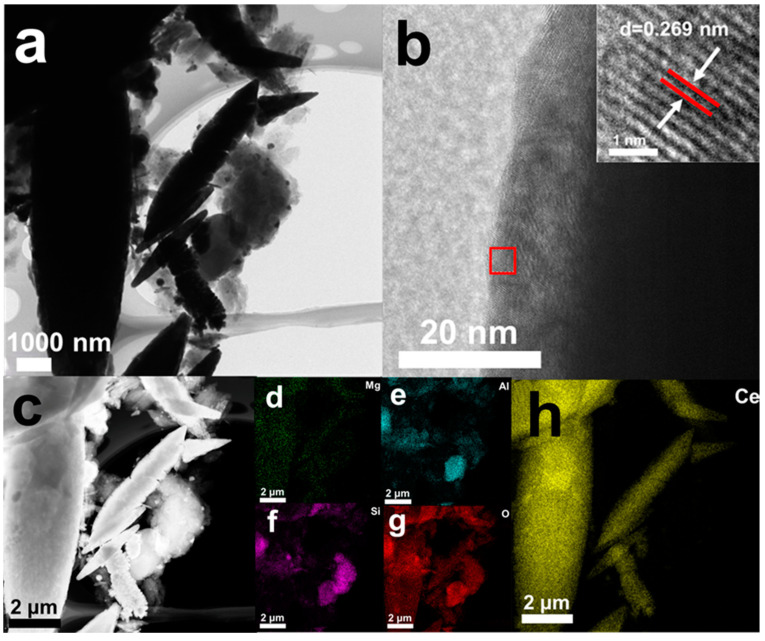
(**a**) TEM image; (**b**) lattice fringing; (**c**) high-definition TEM image; elemental mapping spectra of (**d**) Mg, (**e**) Al, (**f**) Si, (**g**) O, and (**h**) Ce.

As shown in Figure 4, the TEM images of 0.1 g ATP/CeO_2_ revealed a series of sharp rod-like and sheet-like structures, which were typical morphological characteristics of attapulgite (ATP). Owing to its high specific surface area and unique layered structure, ATP was widely used in adsorption and catalysis. These structures provided an extensive surface area that facilitated fluoride ion adsorption. The TEM image in Figure 4a presented a high-resolution view of an individual ATP nanorod, showing detailed structural characteristics. The surface appeared relatively smooth with clear edges, which was beneficial for providing stable reaction sites during the adsorption process. The TEM image in Figure 4c displayed cerium dioxide (CeO_2_) with a smooth, uniform surface and a relatively dense structure. Figure 4d illustrates the distribution of magnesium within the material, indicating a uniform dispersion of this major component of ATP throughout the sample. As shown in Figure 4e, the distribution of aluminum was similar to that of magnesium, demonstrating a uniform dispersion of another key component of ATP. Figure 4f revealed a concentrated distribution of silicon in the sample, indicating the presence of silica, a natural component of ATP. The presence of silicon was particularly important for the structural stability and adsorption properties of the material. As illustrated in Figure 4g, oxygen was widely distributed throughout the sample, indicating the presence of oxides such as ATP and CeO_2_, which were critical for the material’s chemical properties and adsorption reactions. In Figure 4h, Ce is mainly concentrated in specific regions of the shuttle structure, which is consistent with the SEM image of CeO_2_. The presence of Ce may have played a pivotal role in the material’s adsorption performance, particularly for F^−^.

#### 3.1.3. FT-IR Analysis

**Figure 5:**
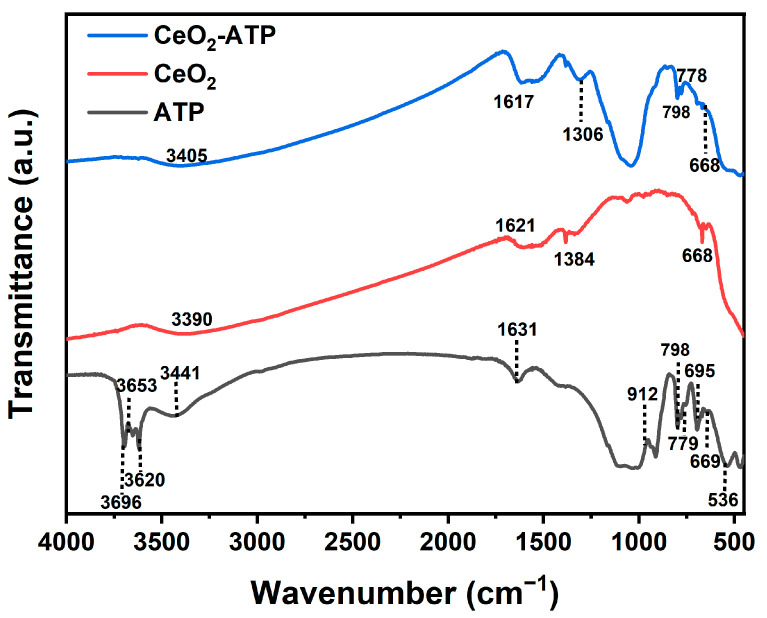
FT-IR images of CeO_2_/ATP, CeO_2_, ATP.

As shown in Figure 5, FT-IR was utilized to analyze ATP, CeO_2_, and CeO_2_/ATP. In the ATP spectrum, the peaks at 3698.57 cm^−1^ and 3629.54 cm^−1^ corresponded to the stretching vibrations of water molecules in the interlayer of attapulgite. The peak observed at 1039.49 cm^−1^ is attributed to the Si-O stretching vibration characteristic of silicate quartz. Peaks in the lower wavenumber region, such as 916.79 cm^−1^ and 844.16 cm^−1^, were likely associated with the deformation vibrations of the Al-Mg-OH layers in attapulgite.

In the CeO_2_ spectrum, the peaks at 1384.03 cm^−1^ and 1619.07 cm^−1^ were mainly attributed to CO_3_^2−^ vibrations, caused by carbonate impurities in the sample. Other weaker peaks, such as 1127.55 cm^−1^, may have resulted from minor impurities introduced during sample preparation or from the environment. The FT-IR spectrum of the CeO_2_/ATP composite exhibited more complex absorption peaks compared to pure attapulgite or cerium dioxide. The peaks at 3690.15 cm^−1^ and 3628.09 cm^−1^ indicated that the intrinsic structure of ATP was preserved throughout the modification process. The newly appeared peaks at 1387.17 cm^−1^ and 1619.07 cm^−1^ suggested that the presence of CeO_2_ may have influenced the carbonate content on the sample surface or other related chemical species. In the wavenumber range from 1000 to 400 cm^−1^, the modified sample displayed some new or intensified peaks, which could have indicated interactions between CeO_2_ and ATP, such as the formation of Ce-O-Si or Ce-O-Al bonds. The addition of CeO_2_ had a noticeable impact on the FT-IR spectrum of attapulgite, indicating certain chemical and physical interactions between CeO_2_ and ATP. These interactions were likely significant for the material’s fluoride ion adsorption performance.

#### 3.1.4. XPS Analysis

**Figure 6:**
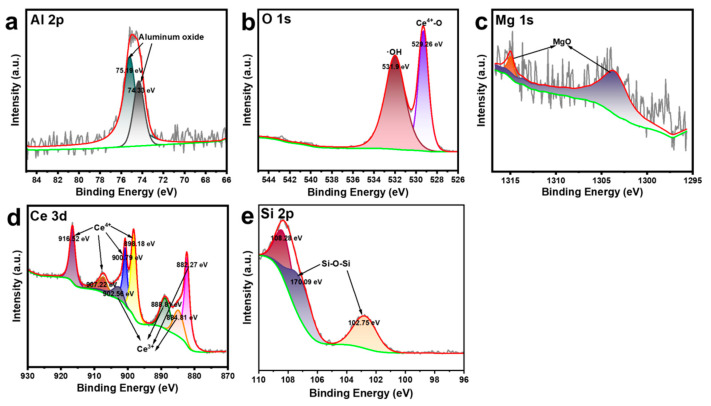
High-resolution XPS spectra of (**a**) Al 2p, (**b**) O 1s, (**c**) Mg 1s, (**d**) Ce 3d and (**e**) Si 2p of CeO_2_/ATP.

As shown in Figure 6, the Al 2p peaks appeared at 75.19 eV and 74.33 eV, consistent with the form of Al in aluminum oxide, indicating the presence of aluminum oxide in the sample. For magnesium oxide (MgO), the binding energies of Mg 1s were observed at 1314.97 eV and 1303.54 eV, which were close to the reported Mg 1s binding energy for MgO (approximately between 1303 eV and 1305 eV). This peak represented the typical oxidation state of magnesium in MgO, which is Mg^2+^. In the lattice structure of MgO, magnesium typically existed in the +2 oxidation state.

The binding energies at 900.79 eV, 898.18 eV, 907.22 eV, and 916.52 eV were typically associated with Ce^4+^, the primary oxidation state in CeO_2_. The lower-energy peaks observed at 882.27 eV, 884.81 eV, 888.81 eV, and 902.56 eV corresponded to Ce^3+^, indicating the presence of a partially reduced state. The binding energy of 108.28 eV indicated that silicon (Si) was in a high oxidation state, likely Si^4+^, which corresponds to a silicate. The binding energies of 102.75 eV and 107.09 eV suggested different forms of silicon, possibly involving Si-O-Si bonds with slight variations in bonding environments. The binding energy at 529.26 eV corresponded to lattice oxygen in CeO_2_ and was typically associated with Ce^4+^–O bonds, representing oxygen in a fully oxidized cerium oxide matrix. The binding energy at 531.9 eV indicated oxygen associated with hydroxyl groups (OH) or surface-adsorbed oxygen species. Such oxygen species may have originated from surface hydroxylation or defect sites, usually related to oxygen vacancies and the presence of Ce^3+^ in cerium oxide.

### 3.2. Fluoride Adsorption

#### 3.2.1. Different Adsorbents

As shown in Figure S3, to further explore the efficiency of F^−^ removal by CeO_2_, ATP, and their composite material CeO_2_/ATP, systematic adsorption experiments were conducted under identical conditions with an initial F^−^ concentration (C_0_) of 10 mg/L and a temperature (T) of 25 °C. The removal rates and adsorption capacities of these materials were measured at different dosages. The results, visually presented in Figure 1, clearly demonstrated the performance differences among the individual adsorbents and the significant advantages of the CeO_2_/ATP composite material.

As a metal oxide with excellent redox properties, CeO_2_ exhibited some capacity for fluoride ion adsorption. However, in this study, the increase in removal rate with higher dosages was relatively limited, possibly owing to the limited number of active sites on the CeO_2_ surface under the experimental conditions employed in this study, ATP did not achieve an ideal fluoride ion removal rate, especially at high dosages, where the increase in removal rate reached a plateau.

Compared to single-component CeO_2_ and ATP, the CeO_2_/ATP composite demonstrated a significant advantage in terms of removal rate. Regarding adsorption capacity, although CeO_2_ and ATP showed some adsorption ability at specific dosages, their overall capacity tended to decrease with increasing dosage. This likely resulted from the gradual saturation of adsorption sites and the reduction in interaction strength between the adsorbent and fluoride ions. The CeO_2_/ATP composite not only maintained a high removal rate but also exhibited relatively stable adsorption capacity. This further confirmed its potential as an efficient fluoride ion adsorbent. The study indicated that the combination of CeO_2_ and ATP not only enhanced the adsorption performance of the material but also optimized its interaction mechanism with fluoride ions.

#### 3.2.2. Different Dosage

As shown in Figure S4, the effect of different adsorbent dosages on the fluoride removal efficiency of the CeO_2_/ATP adsorbent was investigated under conditions of 10 mg/L fluoride ion concentration and a temperature of 25 °C. The experimental results indicated that as the adsorbent dosage was set from 0.1 g/L to 1 g/L, the fluoride removal efficiency increased significantly as a result of the fast bonding between F^−^ ions and the active adsorption sites on the surface of CeO_2_/ATP. In particular, the removal rate was 56.87% better than the initial figure.

This phenomenon could be attributed to two main factors. First, the increase in dosage directly provided more active adsorption sites, enabling each additional site to capture a fluoride ion while the fluoride ion concentration remained constant, thereby enhancing the removal rate. Second, although the increase in dosage introduced more active sites, the adsorption capacity exhibited a downward trend. This was primarily because with a large amount of CeO_2_/ATP adsorbent added, numerous active sites were exposed, but due to the constant fluoride ion concentration, there was insufficient adsorption driving force, leading to some active sites remaining unoccupied. Consequently, there were less fluoride ions adsorbed per mass of adsorbent.

It is noteworthy that at a dose of 0.4 g L^−1^, the CeO_2_/ATP adsorbent achieved optimal fluoride removal efficiency. Moreover, augmenting the dosage further did not substantially influence the adsorption efficiency. From environmental and economic perspectives, it is important to determine the optimal dosage to ensure that the post-adsorption fluoride concentration complies with the permissible limits for drinking water quality standards. Therefore, the optimal dosage of the adsorbent is 0.4 g L^−1^, and this dosage was used for subsequent experiments.

##### 3.2.3. pH

As shown in Figure S5, within the pH range of 0–3, the fluoride adsorption capacity of CeO_2_/ATP increased as pH rose. At pH 3, the adsorption capacity and removal efficiency for fluoride ions reached their peak values of 37.69 mg/g and 96%, respectively. However, as the pH continued to increase beyond this point, adsorption performance declined sharply. Under acidic conditions, the elevated proton concentration in the solution resulted in an increased positive surface charge on the CeO_2_/ATP composite, thereby strengthening the electrostatic interaction with negatively charged fluoride ions and enhancing adsorption efficiency, thereby improving both adsorption efficiency and capacity.

In an acidic environment, certain functional groups on the composite surface underwent protonation, causing changes in the surface structure that exposed more active sites and increased contact opportunities with fluoride ions. Furthermore, the lower concentration of OH^−^ under acidic conditions reduced competition with fluoride ions for adsorption sites, allowing more sites to be available for fluoride capture. At low fluoride concentrations, acidic conditions promoted the formation of stronger chemical bonds between fluoride ions and the surface of the CeO_2_/ATP composite, thereby enhancing adsorption stability and removal efficiency.

This revision maintains the scientific integrity of the information while ensuring that past tense is used consistently throughout the section.

#### 3.2.4. Adsorption Kinetics

**Figure 7:**
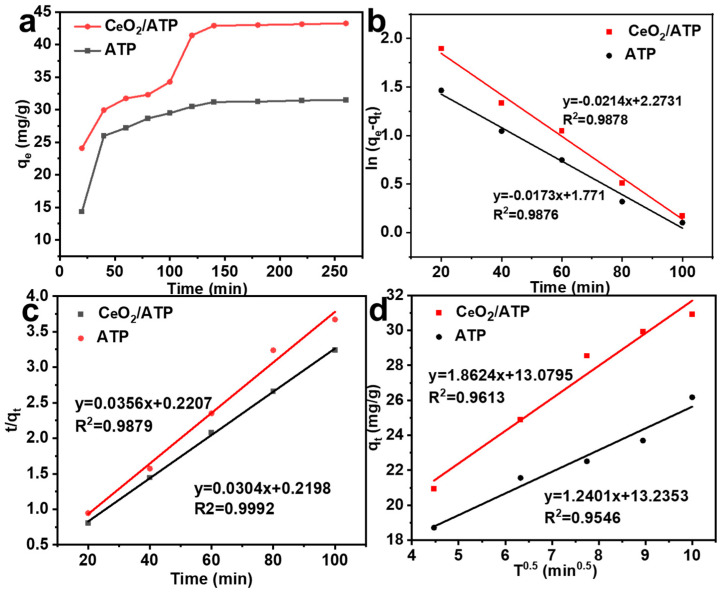
(**a**) Fluoride adsorption kinetics of ATP and CeO_2_/ATP. (**b**) Kinetic model graphs of pseudo-first-order, (**c**) pseudo-second-order, and (**d**) intra-particle diffusion.

As shown in Figure 7a–d, the kinetic models of ATP and CeO_2_/ATP were analyzed using pseudo-first-order, pseudo-second-order, and intra-particle diffusion models to perform linear fitting for the fluoride adsorption experiments. The adsorbent dosage of ATP and CeO_2_/ATP was 0.4 g/L, with T = 298 K, pH = 3, and C_0_(F^−^) = 10 mg/L. The adsorption behavior of ATP and CeO_2_/ATP over time was studied. In the first 40 min, the presence of abundant active sites generated a significant adsorption-driving force, facilitating enhanced uptake capacity.

From 40 to 140 min, the adsorption rate slowed down, and after 140 min, the adsorption capacity gradually reached equilibrium, with a maximum adsorption capacity of 43.27 mg/g for the adsorbent.

As shown in Table 1, when compared with other standard materials, the material prepared in this study exhibited ultra-fast kinetics, demonstrating more efficient adsorption performance than other hydroxyapatite (HAP)-based materials. Equations (1) and (2) provided the linear forms of the pseudo-first-order and pseudo-second-order models, while Equation (3) provided the linear form of the intra-particle diffusion model:

(1) $$\ln\left(q_{e}-q_{t}\right)=lnq_{e}-k_{1}t\;$$

(2) $$\frac{t}{q_{t}}=\frac{1}{k_{2}q_{e}^{2}}+t/q_{e}\;$$

(3) $$q_{t}=k_{3}t^{0.5}+c\;$$

In the fluoride ion adsorption experiments, different kinetic models were employed and represented the fluoride ion adsorption capacity at equilibrium and at a given time, respectively. The pseudo-first-order rate constant 1 was calculated from the slope of a specific linear plot, but the theoretical values did not match the experimental values. In contrast, the pseudo-second-order rate constant 2 was obtained from the slope and intercept of another plot, and the adsorption capacity calculated by this model was more consistent with the experimental values, with a correlation coefficient 2 as high as 0.99. Furthermore, the intra-particle diffusion rate constant (k_3_) and the boundary layer thickness constant indicated that the adsorption process was governed by a combination of external mass transfer and intra-particle diffusion mechanisms, as indicated by two linear regression regions. By fitting and comparing the kinetic models, this study found that the pseudo-second-order kinetic model provided the best fit, indicating that chemisorption was the dominant mechanism.

#### 3.2.5. Adsorption Isotherms

**Figure 8:**
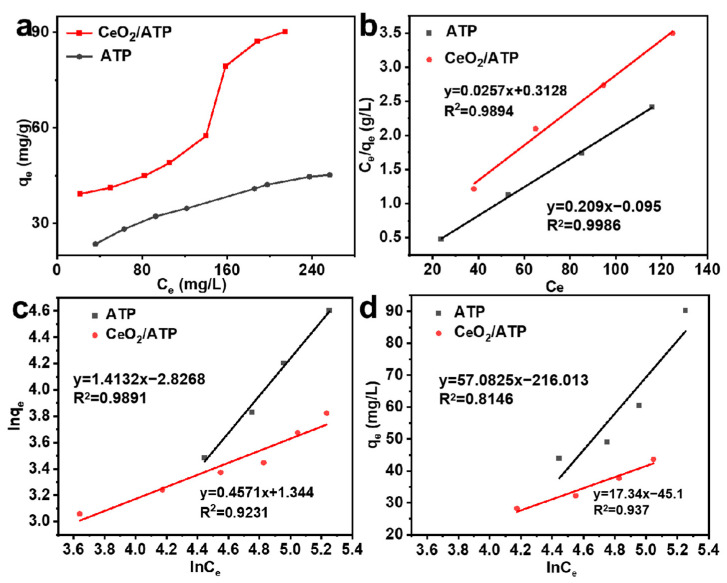
(**a**) Adsorption isotherms of fluorine on the HAP and CeO_2_/HAP samples. (**b**) Langmuir isotherm simulation. (**c**) Freundlich isotherm simulations (**d**) Temkin model of isotherms.

To further investigate the isotherm model for fluoride adsorption by CeO_2_/ATP, the adsorption behavior was systematically analyzed under neutral conditions with initial fluoride concentrations ranging from 10 to 260 mg/L. As shown in Figure 8, the adsorption capacity of CeO_2_/ATP increased significantly with higher initial fluoride concentrations, rising from 39.3 mg/g to 90.24 mg/g. This phenomenon could be attributed to the enhanced adsorption driving force provided by the increased fluoride concentration, which facilitated the adsorption of more fluoride ions onto the material’s surface. The rapid increase in adsorption rate further confirmed the efficient utilization of active sites on the CeO_2_/ATP surface. Notably, under all tested conditions, the adsorption performance of CeO_2_/ATP was significantly superior to that of pure ATP, due to the higher specific surface area of CeO_2_/ATP, which provided a greater number of adsorption sites.

The adsorption isotherm equations used were as follows:

(4) $$\mathrm{Langmuir}:\;\frac{c_{e}}{q_{e}}=\frac{1}{k_{L}q_{m}}+\frac{c_{e}}{q_{m}}$$

(5) $$\mathrm{Freundlich}:\;lnq_{e}=lnk_{F}+\frac{lnc_{e}}{n}$$

(6) $$\mathrm{Temkin}:\;q_{e}=blna+blnc_{e}$$

In these equations, (mg/g) represents the amount of fluoride adsorbed at equilibrium, and (mg/L) denotes the equilibrium concentration of fluoride ions. (mg/g) indicates the theoretical maximum adsorption capacity, while (L/mg) is the Langmuir constant related to adsorption energy. (mg/g) is the Freundlich constant associated with the adsorption capacity of the adsorbent, and 1/ represents the adsorption intensity.

Figure 8b–d presented the isotherm model fitting results for fluoride adsorption, with the relevant parameters shown in Table 2. The adsorption data for both ATP and CeO_2_/ATP fit the Langmuir model well (R^2^ ≥ 0.990), indicating monolayer adsorption characteristics. The maximum adsorption capacities of ATP and CeO_2_/ATP were determined to be 38.9 mg/g and 47.84 mg/g, respectively, suggesting that CeO_2_/ATP outperformed most adsorbents and had potential for treating fluoride-containing wastewater. The Langmuir model provided a closer representation of the actual adsorption process. The Temkin model showed correlation coefficients of 0.8146 and 0.9371 for ATP and CeO_2_/ATP, respectively, indicating that chemisorption played a dominant role.

### 3.3. Coexisting Ions

**Figure 9:**
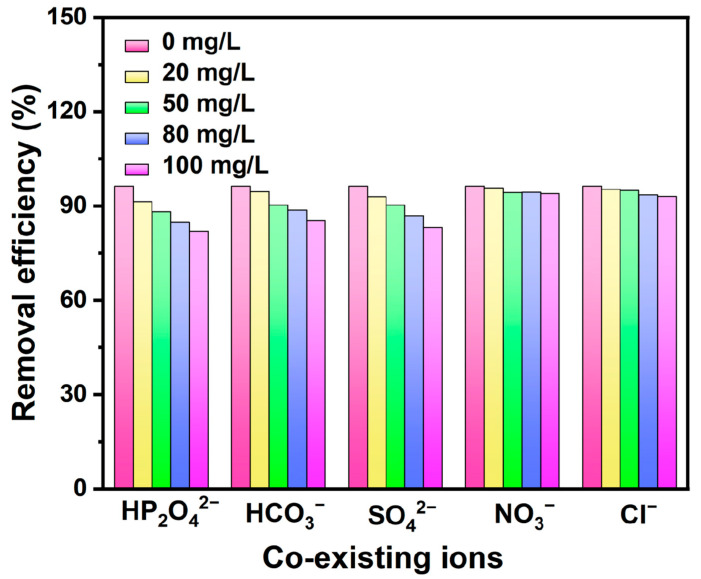
Effect of coexisting ions on adsorbed fluorine.

As shown in Figure 9, natural waters such as lake water, river water, groundwater, and industrial wastewater typically contain various anions, including but not limited to nitrates, chlorides, carbonates, sulfates, and phosphates. These coexisting anions could interfere with fluoride adsorption by competing for active sites. To investigate this effect in detail, adsorption experiments were conducted with CeO_2_/ATP, setting the concentration of coexisting anions at 2, 5, 8, and 10 times the concentration of fluoride ions. The results indicated that, owing to the positive charge present on the surface of CeO_2_/ATP, certain anions could be effectively adsorbed via electrostatic interactions. To be specific, the greater the concentration of anions is, the more significant the negative impact on the adsorption of fluoride ions will be. Notably, the impact of coexisting anions on fluoride adsorption varied depending on their valency and properties. Low-valency anions, such as NO_3_^−^ and Cl^−^, exhibited a slight promoting effect on fluoride adsorption under certain conditions. This may have been due to the relatively weak electrostatic interactions between these anions and the positively charged CeO_2_/ATP surface, which reduced their competition for fluoride adsorption sites to some extent.

However, high-valency anions, such as HP_2_O_4_^2−^ and SO_4_^2−^, had a negative impact on fluoride adsorption. This could have been because these anions had higher electron density, leading to stronger electrostatic interactions with the positively charged CeO_2_/ATP surface, thereby occupying more adsorption sites and reducing the adsorbent’s capacity for fluoride ions. In addition to electrostatic interactions, coexisting anions may have also reduced the overall adsorption capacity by occupying adsorption sites. Based on the experimental results, the impact of the coexisting anions on the adsorption of fluoride was arranged in the following order: HP_2_O_4_^2−^ > SO_4_^2−^ > HCO_3_^−^ > Cl^−^ > NO_3_^−^.

### 3.4. Recyclability

**Figure 10:**
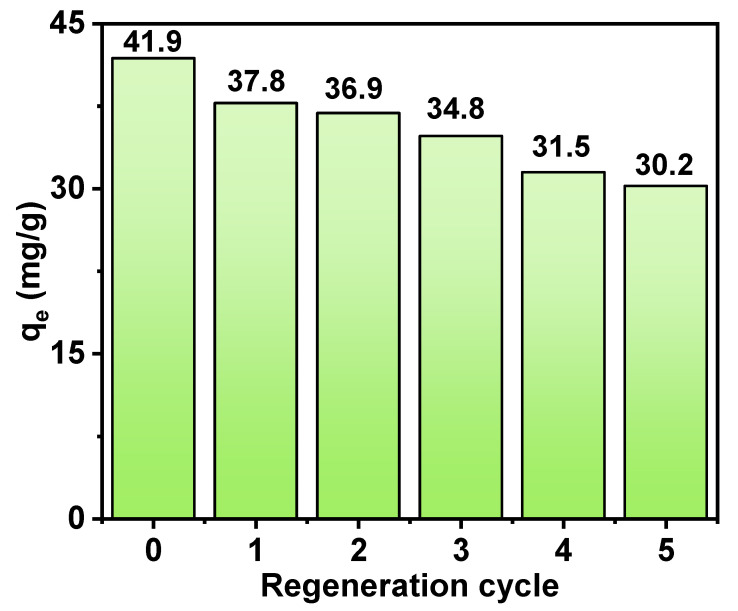
Cyclic adsorption and desorption of CeO_2_/ATP.

As shown in Figure 10, reusability was a key indicator of the overall performance of an adsorbent and was crucial for evaluating its economic and environmental viability in practical applications. To thoroughly investigate the reusability of CeO_2_/ATP, a series of five consecutive adsorption-desorption cycles was conducted using 0.5 mol/L NaOH and 0.5 mol/L Na_2_CO_3_ solutions as desorption agents.

The findings indicated that following the first cycle, the adsorption capacity of CeO_2_/ATP attained 37.8 mg/g, which demonstrated its favorable initial adsorption property. However, with each successive cycle, the adsorption capacity gradually decreased, dropping to 36.9 mg/g in the second cycle, 34.8 mg/g in the third cycle, 31.5 mg/g in the fourth cycle, and finally to 30.2 mg/g in the fifth cycle. This trend suggested that while CeO_2_/ATP had certain reusability potential, its adsorption performance deteriorated over continuous cycles.

This decline in capacity could have been owing to the incomplete regeneration of active sites on the CeO_2_/ATP surface after desorption treatment with the NaOH-Na_2_CO_3_ solution. These active sites served as vital interaction spots with the adsorbate throughout the adsorption procedure, and the extent of their regeneration directly affected the adsorbent’s subsequent adsorption performance. Consequently, partially unrecovered active sites could have led to the gradual decrease in adsorption capacity. Nevertheless, the results of the cyclic adsorption-regeneration test confirmed that CeO_2_/ATP, as a reusable adsorbent, held promise for practical applications, providing certain economic and environmental benefits.

### 3.5. Fluoride Removal Mechanism

The CeO_2_/ATP composite material demonstrates superior performance in fluoride removal, primarily due to its distinctive surface positive charge characteristics, intricate structure, and the synergistic effects of multiple adsorption mechanisms. The positive charge originates mainly from Ce^4+^ ions in CeO_2_ and Mg and Al atoms in ATP. The presence of Ce^4+^ imparts a strong positive charge to the material’s surface, creating a favorable electrostatic environment for fluoride ion adsorption. Because of the strong contact between the negatively charged fluoride ions and the positively charged CeO_2_/ATP surface, electrostatic adsorption is the main mechanism that propels fluoride ion attachment to the material surface. Additionally, the ionic radius of fluoride ions is comparable to that of oxygen ions in the CeO_2_ lattice, enabling fluoride ions to replace oxygen ions via ion exchange, thereby enhancing adsorption efficiency. During the adsorption process, the regulation of surface charge balance plays a crucial role. The adsorption of fluoride ions disrupts surface charge equilibrium, promoting the reduction of Ce^4+^ to Ce^3+^ and releasing additional electrons. These electrons aid in the creation of oxygen vacancies, which serve as fresh adsorption locations and enhance the material’s ability to adsorb fluoride ions.

Compared to pure ATP and CeO_2_, the CeO_2_/ATP composite material offers significant advantages in fluoride removal. While pure ATP possesses some adsorption capacity, its limited specific surface area and fewer adsorption sites, coupled with the absence of electrostatic adsorption and ion exchange mechanisms provided by CeO_2_, result in weaker fluoride removal performance. Although CeO_2_ has a large specific surface area and electrostatic adsorption capability, it lacks the complex pore structure and ion exchange mechanisms of ATP, leading to inferior fluoride removal compared to the composite material. By integrating the benefits of both CeO_2_ and ATP, the CeO_2_/ATP composite material achieves a higher specific surface area, more adsorption sites, and multiple adsorption mechanisms, making it an efficient, stable, and selectively fluoride-ion-adsorbent.

In summary, the CeO_2_/ATP composite material achieves highly effective fluoride ion adsorption through its unique surface properties, complex structure, and synergistic adsorption mechanisms. These insights not only provide a theoretical foundation for its application in fluoride removal but also offer valuable references for developing novel adsorption materials. In practical water treatment applications, the CeO_2_/ATP composite material shows great potential as an effective solution for addressing fluoride pollution.

## 4. Conclusions

This study used a straightforward hydrothermal approach to successfully synthesis a CeO_2_/ATP composite, which was then used to remove fluoride from water. The mechanisms involved in fluoride removal were thoroughly analyzed. The maximum adsorption capacity of CeO_2_/ATP was determined to be 47.84 mg/g and it outperformed most adsorbents and had potential for treating fluoride-containing wastewater. The resulting composite demonstrated ultra-fast fluoride removal kinetics, achieving adsorption equilibrium within 160 min, with the kinetics conforming to a pseudo-second-order model. The primary mechanisms for fluoride ion removal by the CeO_2_/ATP adsorbent included ion exchange, surface adsorption, and electrostatic attraction. Overall, the findings of this study indicate that CeO_2_/ATP nanocomposites are highly effective and promising adsorbents for fluoride removal, providing a viable solution for addressing fluoride contamination in water treatment applications.

## Figures and Tables

**Table 1:** Adsorption kinetic parameters of the pseudo-first-order and pseudo-second-order kinetic models.

Sample	Pseudo-First-Order			Pseudo-Second-Order			Intragranular Diffusion Model	
	K_1_	q_e_	R_2_	K_2_	q_e_	R^2^	K_3_	R^2^
ATP	0.0214	5.88	0.9878	0.0356	28.09	0.9879	1.2401	0.9546
CeO_2_/ATP	0.0173	9.71	0.9876	0.0304	32.89	0.9992	1.8624	0.9613

**Table 2:** Parameters of the Langmuir, Freundlich, and Temkin isotherm models for fluoride adsorption.

Sample	Langmuir			Freundlich			Temkin		
	q_max_	K_L_	R^2^	K_F_	n	R^2^	b	a	R^2^
ATP	38.9	12.17	0.9979	16.891	0.7076	0.9891	57.0825	44.002	0.8146
CeO_2_/ATP	47.84	0.4546	0.9978	3.83	0.4571	0.9231	17.3453	13.532	0.9371

## Data Availability

Data are contained within the article and Supplementary Materials.

## Supplementary Materials

The following supporting information can be downloaded at: https://www.mdpi.com/article/10.3390/nano15050357/s1, Figure S1: (a,b) SEM images of ATP at different magnifications, (c) elemental mapping spectra, elemental mapping spectra of (d) O, (e) Al, (f) Mg, and (g) Si. Figure S2: (a,b) SEM images of CeO_2_ at different magnifications, (c) elemental mapping spectra, elemental mapping spectra of (d) Ce and (e) O, (f) EDS energy. Figure S3: Adsorption capacity and removal rate of three adsorbents. Figure S4: Effect of different dosages on adsorption experiments. Figure S5: Effect of different pH on adsorption experiments. Table S1: Comparison of fluoride adsorption capacities of various adsorbents reported in the literature.

## References

[1] Ngantcha-Kwimi T.A., Brian E.R. (2016). As(V) and PO_4_ Removal by an Iron-Impregnated Activated Carbon in a Single and Binary Adsorbate System: Experimental and Surface Complexation Modeling Results. J. Environ. Eng..

[2] Biswas G., Kumari M., Adhikari K., Dutta S. (2017). A Critical Review on Occurrence of Fluoride and Its Removal through Adsorption with an Emphasis on Natural Minerals. Curr. Pollut. Rep..

[3] Biswas K., Saha S.K., Ghosh U.C. (2007). Adsorption of Fluoride from Aqueous Solution by a Synthetic Iron(III)−Aluminum(III) Mixed Oxide. Ind. Eng. Chem. Res..

[4] Dhillon A., Soni S.K., Kumar D. (2017). Enhanced fluoride removal performance by Ce–Zn binary metal oxide: Adsorption characteristics and mechanism. J. Fluorine Chem..

[5] Fan Q.H., Tan X.L., Li J.X., Wang X.K., Wu W.S., Montavon G. (2009). Sorption of Eu(III) on Attapulgite Studied by Batch, XPS, and EXAFS Techniques. Environ. Sci. Technol..

[6] Fu M., Li X., Jiang R., Zhang Z. (2018). One-dimensional magnetic nanocomposites with attapulgites as templates: Growth, formation mechanism and magnetic alignment. Appl. Surf. Sci..

[7] García-Sánchez J.J., Solache-Ríos M., Martínez-Gutiérrez J.M., Arteaga-Larios N.V., Ojeda-Escamilla M.C., Rodríguez-Torres I. (2016). Modified natural magnetite with Al and La ions for the adsorption of fluoride ions from aqueous solutions. J. Fluorine Chem..

[8] Ghazy O.A., Khalil S.A., Senna M.M. (2020). Synthesis of montmorillonite/chitosan/ammonium acrylate composite and its potential application in river water flocculation. Int. J. Biol. Macromol..

[9] Ghosh S., Malloum A., Igwegbe C.A., Ighalo J.O., Ahmadi S., Dehghani M.H., Othmani A., Gökkuş Ö., Mubarak N.M. (2022). New generation adsorbents for the removal of fluoride from water and wastewater: A review. J. Mol. Liq..

[10] Inaniyan M., Raychoudhury T. (2019). Application of activated carbon–metal composite for fluoride removal from contaminated groundwater in India. Int. J. Environ. Sci. Technol..

[11] Li Y., Shi M., Xia M., Wang F. (2021). The enhanced adsorption of Ampicillin and Amoxicillin on modified montmorillonite with dodecyl dimethyl benzyl ammonium chloride: Experimental study and density functional theory calculation. Adv. Powder Technol..

[12] Lin J., Wu Y., Khayambashi A., Wang X., Wei Y. (2017). Preparation of a novel CeO_2_/SiO_2_ adsorbent and its adsorption behavior for fluoride ion. Adsorpt. Sci. Technol..

[13] Liu B., Liu J., Li T., Zhao Z., Gong X.-Q., Chen Y., Duan A., Jiang G., Wei Y. (2015). Interfacial Effects of CeO_2_-Supported Pd Nanorod in Catalytic CO Oxidation: A Theoretical Study. J. Phys. Chem. C.

[14] Liu H., Deng S., Li Z., Yu G., Huang J. (2010). Preparation of Al–Ce hybrid adsorbent and its application for defluoridation of drinking water. J. Hazard. Mater..

[15] Liu W., Wang D., Soomro R.A., Fu F., Qiao N., Yu Y., Wang R., Xu B. (2019). Ceramic supported attapulgite-graphene oxide composite membrane for efficient removal of heavy metal contamination. J. Membr. Sci..

[16] Ma Z., Zhang Q., Weng X., Mang C., Si L., Guan Z., Cheng L. (2017). Fluoride ion adsorption from wastewater using magnesium(II), aluminum(III) and titanium(IV) modified natural zeolite: Kinetics, thermodynamics, and mechanistic aspects of adsorption. J. Water Reuse Desalin..

[17] Meenakshi S., Sundaram C.S., Sukumar R. (2008). Enhanced fluoride sorption by mechanochemically activated kaolinites. J. Hazard. Mater..

[18] Merodio-Morales E.E., Reynel-Ávila H.E., Mendoza-Castillo D.I., Duran-Valle C.J., Bonilla-Petriciolet A. (2020). Lanthanum- and cerium-based functionalization of chars and activated carbons for the adsorption of fluoride and arsenic ions. Int. J. Environ. Sci. Technol..

[19] Yin H., Yan X., Gu X. (2017). Evaluation of thermally-modified calcium-rich attapulgite as a low-cost substrate for rapid phosphorus removal in constructed wetlands. Water Res..

[20] Zhu T., Zhu T., Gao J., Zhang L., Zhang W. (2017). Enhanced adsorption of fluoride by cerium immobilized cross-linked chitosan composite. J. Fluorine Chem..

[21] Zúñiga-Muro N.M., Bonilla-Petriciolet A., Mendoza-Castillo D.I., Reynel-Ávila H.E., Tapia-Picazo J.C. (2017). Fluoride adsorption properties of cerium-containing bone char. J. Fluorine Chem..

