# Supplementary material for: Efficient Fluoride Removal Using a CeO2/Attapulgite (ATP) Composite
Source: Nanomaterials (Basel). 2025 Feb 26;15(5):357. doi: 10.3390/nano15050357 (PMC11901975; doi:10.3390/nano15050357)
Supplement: Supplementary file 1 [file nanomaterials-15-00357-s001.zip › nanomaterials-3473367-supplementary.pdf]

Efficient Fluoride Removal Using a CeO_2/Attapulgite (ATP) Composite

Jianguo Zhu [1], Yeting Chen [1] and Xin Xiao [2,*]

[1] Institute of Pharmaceutical and Biomaterials, Lianyungang Normal College, Lianyungang 222006, China; zjgzz117@163.com (J.Z.); 13851266965@163.com (Y.C.)
[2] Jiangsu Key Laboratory of Function Control Technology for Advanced Materials, Jiangsu Ocean University, Lianyungang 222005, China
* Correspondence: xiaoxin@njust.edu.cn

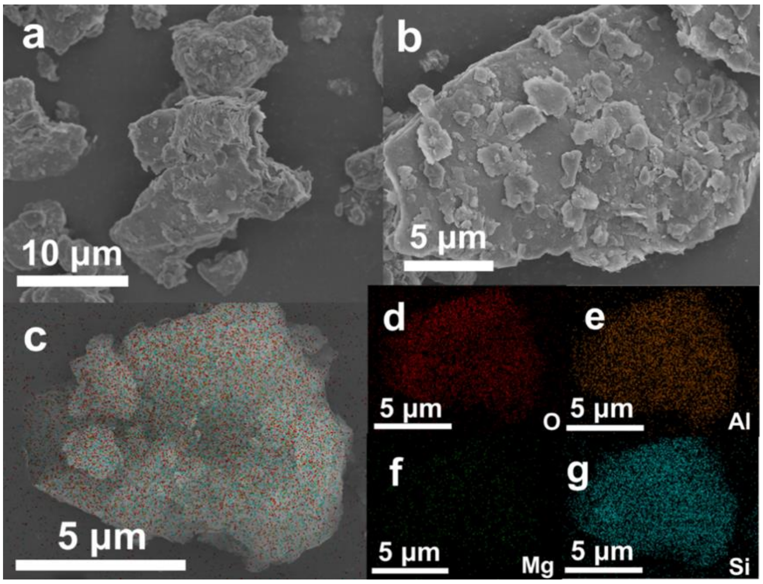

Figure S1 (a,b) SEM images of ATP at different magnifications, (c) elemental mapping spectra, elemental mapping spectra of (d) O, (e) Al, (f)Mg, and (g) Si.

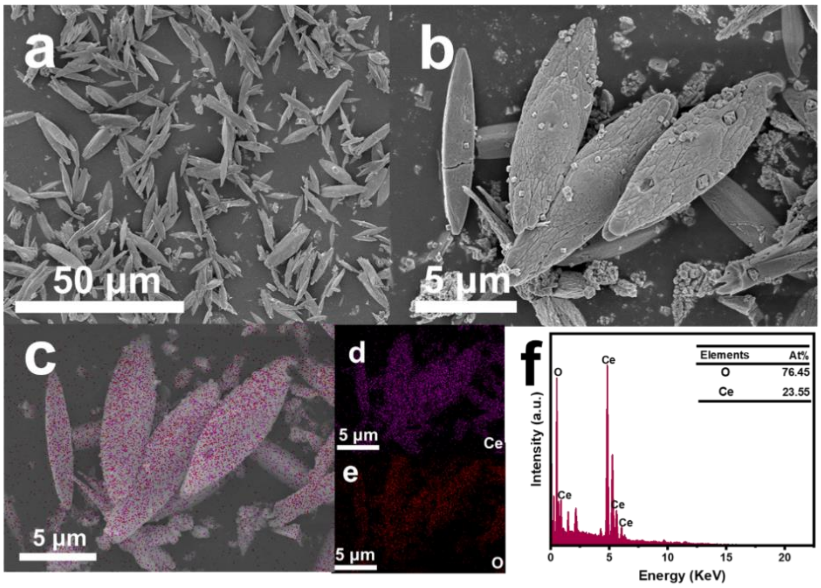

Figure S2 (a,b) SEM images of CeO_2 at different magnifications, (c) elemental mapping spectra, elemental mapping spectra of (d) Ce and (e) O, (f) EDS energy spectra.

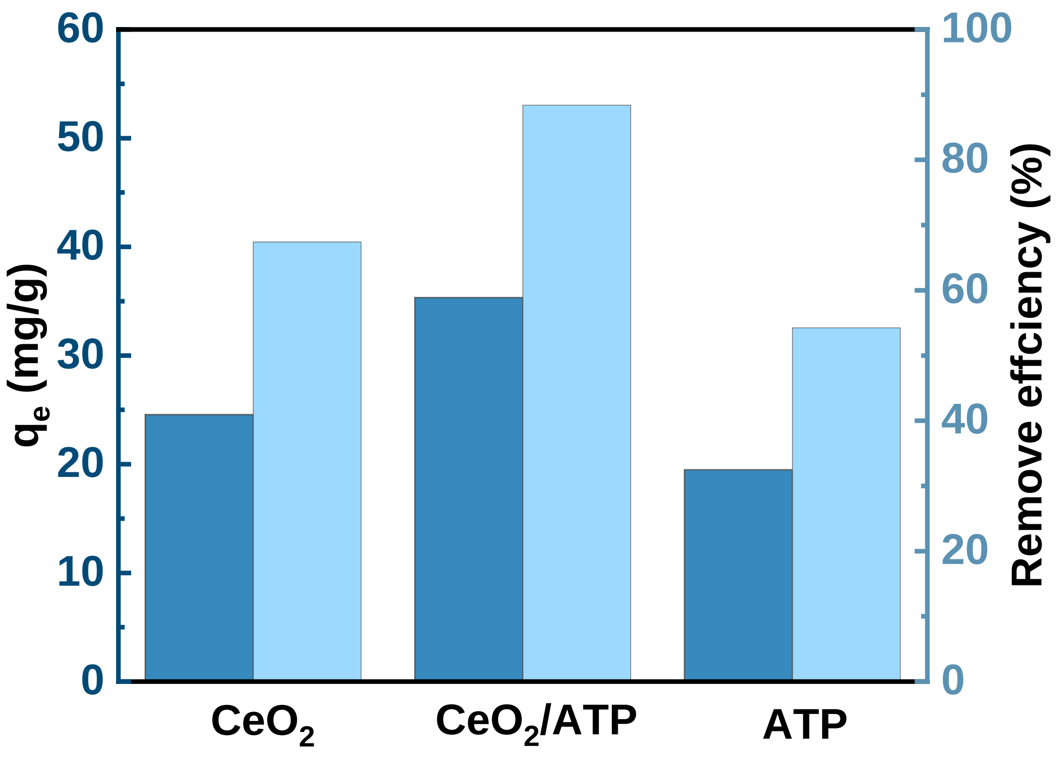

Figure S3 Adsorption capacity and removal rate of three adsorbents.

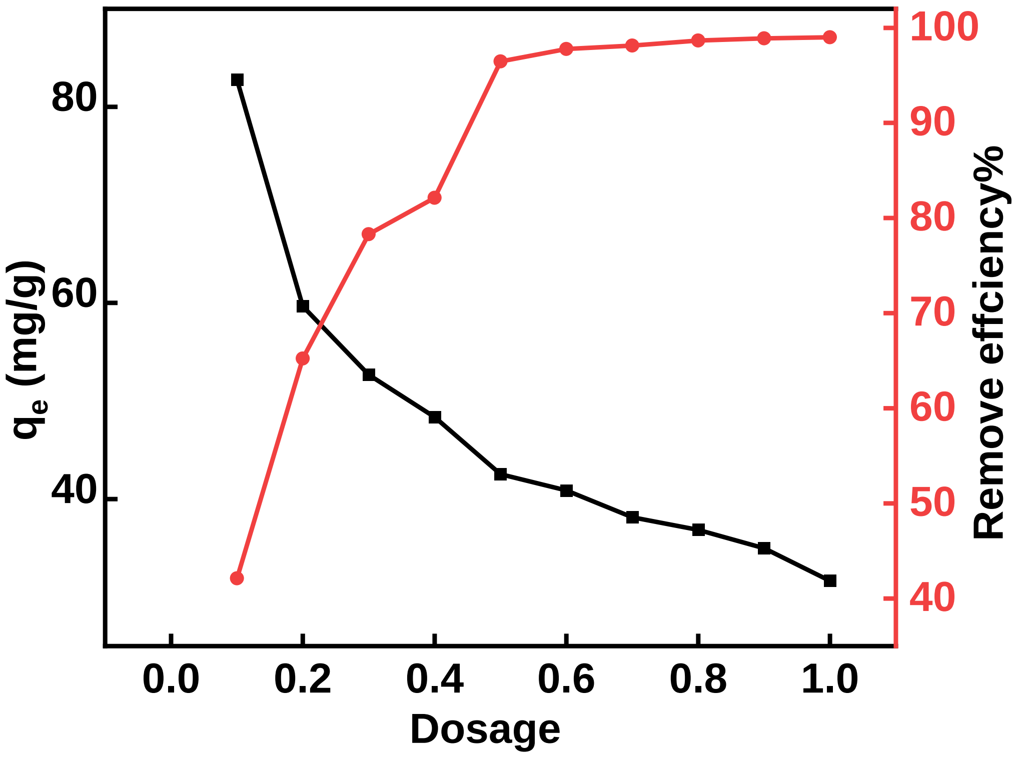

Figure S4 Effect of different dosages on adsorption experiments

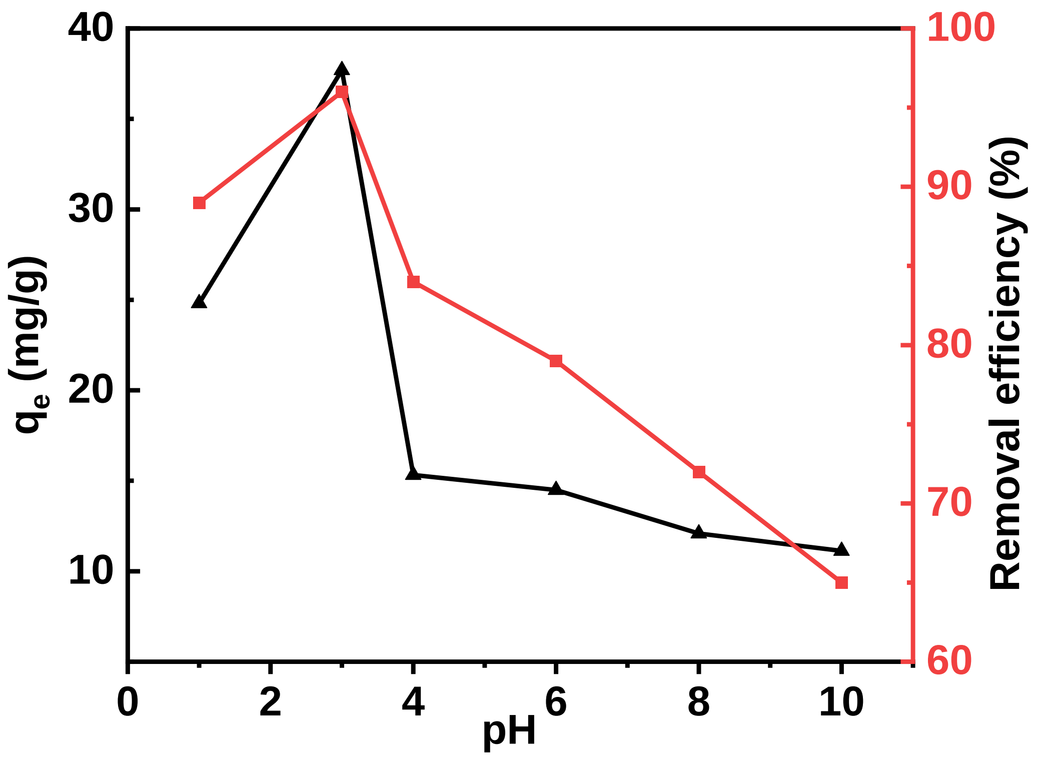

Figure S5 Effect of different pH on adsorption experiments

Table. S1 Comparison of fluoride adsorption capacities of various adsorbents reported in the literature.

Material	Advantages	Disadvantages	References
CeO_2/ATP	Stable material structure with good synergy, simple process, low material cost. The maximum adsorption capacity can reach 47.84 mg/g.	Affected by high-concentration anions with high valence states.	This work
Bi-Fe-HA	The experimental adsorption capacity reaches 54.56 mg/g. Synthesized by the coprecipitation method, with simple operation.	The material cost is relatively higher than that in this study, and the solution pH has a significant impact on the adsorption efficiency of fluoride ions.	0
Green Coconut peel	Widely sourced and low in cost, with good environmental benefits.	The overall adsorption capacity and removal efficiency of the material for fluoride ions are relatively low.	0
modified NaA zeolite	Low material cost, wide application range, and capable of dealing with different water qualities. At a low adsorbent dosage, the maximum adsorption capacity is relatively high.	The synthesis process involves multiple steps such as hydrothermal reaction and the preparation of colloidal hydrated aluminum oxide, and the process is relatively complex.	0

References

1. Adamu, D.B.; Tufa, L.T.; Lee, J.; Zereffa, E.; Segne, T.A.; Razali, M.H. Facile synthesis of bismuth and iron co-doped hy-droxyapatite nanomaterials for high-performance fluoride ions adsorption. J. Environ. Chem. Eng. 2023, 11, 111196.

2. Canciam, C.A.; Pereira, N.C. Assessment of the Use of Epicarp and Mesocarp of Green Coconut for Removal of Fluoride Ions in Aqueous Solution. Int. J. Chem. Eng. 2019, 2019, 7163812.

3. Naskar, M.K. Preparation of colloidal hydrated alumina modified NaA zeolite derived from rice husk ash for effective removal of fluoride ions from water medium. J. Asian Ceram. Soc. 2020, 8, 437–447.
